# Extension of the Measurable Wavelength Range for a Near-Infrared Spectrometer Using a Plasmonic Au Grating on a Si Substrate

**DOI:** 10.3390/mi10060403

**Published:** 2019-06-17

**Authors:** Yu Suido, Yosuke Yamamoto, Gaulier Thomas, Yoshiharu Ajiki, Tetsuo Kan

**Affiliations:** 1 Department of Mechanical Engineering and Intelligent Systems, Graduate School of Informatics and Engineering, The University of Electro-Communications, 1-5-1 Chofugaoka, Chofu-city, Tokyo 182-8585, Japan; suido@ms.mi.uec.ac.jp (Y.S.); yamamoto@ms.mi.uec.ac.jp (Y.Y.); yoshiharu_ajiki@ot.olympus.co.jp (Y.A.); 2 École Nationale Supérieure de Mécanique et des Microtechniques, 26 Rue de l’Épitaphe, 25000 Besançon, France; thomas.gaulier@ens2m.org; 3 Mobile System Development Division, Imager & Analog LSI Technology Department, Olympus Corporation, 2-3 Kuboyama-cho, Hachioji-city, Tokyo 192-8512, Japan

**Keywords:** near-infrared, spectroscopy, surface plasmon resonance, Schottky barrier, grating, Si

## Abstract

In this paper, we proposed near-infrared spectroscopy based on a Si photodetector equipped with a gold grating and extended the measurable wavelength range to cover 1200–1600 nm by improving a spectrum derivation procedure. In the spectrum derivation, photocurrent data during alteration of the incidence angle of the measured light were converted using a responsivity matrix ***R***, which determines the spectroscopic characteristics of the photodetector device. A generalized inverse matrix of ***R*** was used to obtain the spectrum and to fit a situation where multiple surface plasmon resonance (SPR) peaks appeared in the scanning range. When light composed of two wavelengths, 1250 nm and 1450 nm, was irradiated, the two wavelengths were distinctively discriminated using the improved method.

## 1. Introduction

Near-infrared (NIR) spectroscopy is a method of analyzing an object by its optical spectrum, and this method is used in many fields, such as agriculture, chemistry, and medicine [1,2,3,4]. In recent years, compact near-infrared spectrometers have been intensively studied. Particularly, plasmonic-based spectrometers are attracting attention because dispersal of the incident light is performed with a thin layer to allow a compact optical system [5,6,7,8,9]. One such example is based on a plasmonic color filter where the transmission wavelength can be selected by designing periodic structures of a plasmonic metal [6,7,8]. The transmitted light is usually measured by a photodetector located below the plasmonic filter. In addition, an NIR spectrometer using a plasmonic grating on silicon is reported [9]. Since a Schottky barrier is formed at an interface of the grating metal/silicon (Si), coupled surface plasmon resonance (SPR) on the grating is directly detected as a current on the device. The photodetector is integrated with a plasmonic grating, thus this method is well suited for constructing compact NIR spectrometers. However, there is an issue with the Schottky-type spectrometer: The operating wavelength range is only as small as 100 nm. It is therefore required to widen the measurable wavelength range to cover the whole NIR wavelength range for this spectrometer to become practical.

In this paper, we increased the NIR spectroscopy range from 1200 nm to 1600 nm based on a Si photodetector equipped with a gold grating by improving the spectrum derivation procedure.

Although a basic spectrum derivation procedure is provided in a previous reference [9], the simple application of this procedure for a wider NIR range resulted in artifacts in the calculated results due to the multiple SPR coupling points that appeared in the angular photocurrent spectrum. We thus revised the spectrum derivation method to be applicable for a wider NIR wavelength range. We fabricated a plasmonic grating Si photodetector and integrated it with a measurement circuit for low signal noise measurement. The electrical characteristics and SPR photodetection performance of the device were investigated. The spectroscopic performance was evaluated by irradiating the device with light composed of two NIR wavelengths, and the spectroscopic results are shown in the figures below.

## 2. Principles of Light Detection Using Surface Plasmon Resonance (SPR)

**Figure 1:**
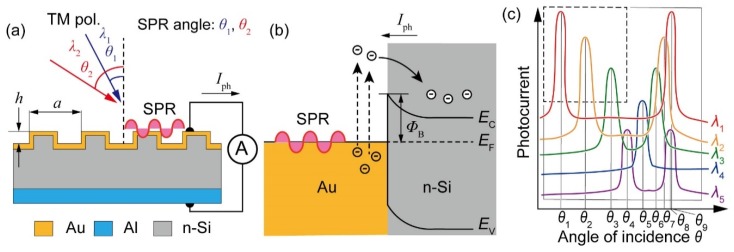
Proposed spectrometer configuration. (**a**) A structure of the photodetector device. (**b**) A current detection mechanism of incident light using surface plasmon resonance (SPR). (**c**) Angular photocurrent spectra for four different wavelength lights.

The structure of the proposed spectrometer is shown in Figure 1a, which was almost the same as that in a previous report [9]. The device consisted of an n-type silicon substrate and a one-dimensional diffraction grating of a thin gold film formed on the n-type silicon substrate. The grooves of the diffraction grating running from the front to the back side of the paper surface are shown in Figure 1a. The gold film also served as the anode electrode. The surrounding medium around the gold grating was assumed to be air. An aluminum film was formed at the bottom surface of the device and served as a cathode electrode.

SPR was excited on the gold grating surface by the light incidence on the grating surface at the resonant incidence angle *θ*, which was dependent on the wavelength *λ* of the light. The SPR coupling behavior was as follows: When transverse magnetic (TM) light with wavelength *λ* entered the grating surface with an incidence angle *θ*, the incident light was diffracted by the grating. At a certain angle of incidence *θ*, the following SPR condition was satisfied, and SPR can be excited on the gold grating surface [10],

(1) $$\frac{\omega }{c}\sqrt{\varepsilon_{\mathrm{air}}}\sin\theta +\frac{2m\pi }{a}=\frac{\omega }{c}\sqrt{\frac{\varepsilon_{\mathrm{air}}\varepsilon_{\mathrm{Au}}}{\varepsilon_{\mathrm{air}}+\varepsilon_{\mathrm{Au}}}},$$

where *ω* is the angular frequency of the incident light, *c* is the speed of light in a vacuum, *m* is the order of diffraction, and *ε*_air_ and *ε*_Au_ are the relative permittivity of air and gold, respectively. The left side of Equation (1) is the wavenumber component along the device surface of the diffracted light. The right side is the wavenumber of SPR on the gold/air interface. SPR was excited on the gold surface when the angular frequency and wavenumber of the incident light and SPR coincided with each other due to the effect of diffraction. Based on this relationship, it can be confirmed that SPR occurred at different angles *θ* if the wavelength *λ* was different. In addition, since the gold grating was formed on the n-type silicon, a Schottky barrier was formed at the interface between gold and n-type silicon [11,12,13,14]. Normally, a Schottky barrier with a height *Φ*_B_ of 0.7–0.8 eV (detection limit wavelength approximately 1.55–1.77 μm) was formed there (Figure 1b) [15]. Free electrons excited by the SPR overcame the barrier and flowed from the gold grating to the n-type silicon, and the current *I*_ph_ had a peak value there.

Figure 1c shows schematic photocurrent *I*_ph_ plots with respect to the angle of incidence *θ* for five different wavelength lights, from *λ*_1_ through *λ*_5_. In this plot, the device was tilted to alter the angle of incidence *θ*. The photocurrent peaks corresponded to situations where Equation (1) was satisfied. The peak’s angular position thus shifted when the wavelength was changed. Assuming that *λ*_1_ < *λ*_2_ < *λ*_3_, the angular positions monotonically shifted from *θ*_1_ through *θ*_2_ to *θ*_3_, as depicted within a dotted line square in Figure 1c. This angular shift direction, plus or minus, is determined by a sign of the corresponding diffraction order *m*. Since the resonant angular positions are determined by the wavelength, the spectral information can be derived using the data plots. This measurement was performed only on a device surface, so the proposed method did not require an optical path to disperse the light.

## 3. Spectral Calculation Procedures

The spectral calculation basically followed the procedures presented in Reference [9]. We first presented the basic procedures and expanded them for a wider range of wavelengths. To calculate the spectrum, we first experimentally derived the light detection sensitivity of the photodetector called the responsivity *R*, which expresses the conversion efficiency from the input light intensity *P*_in_ into a measured photocurrent *I*_ph_, defined as

(2) $$R=\frac{I_{\mathrm{ph}}}{P_{\mathrm{in}}}$$

Since the photocurrent is measured during *θ* alteration, the responsivity ***R*** can be determined at each angle of incidence *θ* for monochromatic light irradiation as $R_{\lambda \theta }=I_{\theta }/P_{\lambda }$, where *λ* is the wavelength of the light. When light composed of several monochromatic lights of different wavelengths is simultaneously illuminated, the generated photocurrent is assumed to be a linear summation of the photocurrent responses of each wavelength component. Assuming that only three different wavelength lights, *λ*_1_, *λ*_2_, and *λ*_3_, as in Figure 1c, are incident on the photodetector, the photocurrent measured at the SPR angle for *λ*_1_, i.e., *θ*_1_, becomes $I_{\theta_{1}}=R_{\lambda_{1}\theta_{1}}P_{\lambda_{1}}+R_{\lambda_{2}\theta_{1}}P_{\lambda_{2}}+R_{\lambda_{3}\theta_{1}}P_{\lambda_{3}}$. The measured value includes contributions from two other light wavelengths, *λ*_2_ and *λ*_3_. Under the same irradiation conditions, if the photocurrents were measured at two other SPR angles of incidence *θ*_2_ and *θ*_3_, the relationship between the photocurrent and incident intensity is expressed in a matrix form as,

(3) $$\left[\begin{array}{c} I_{\theta_{1}}\\ I_{\theta_{2}}\\ I_{\theta_{3}} \end{array}\right]=\left[\begin{array}{ccc} R_{\lambda_{1}\theta_{1}} & R_{\lambda_{2}\theta_{1}} & R_{\lambda_{3}\theta_{1}}\\ R_{\lambda_{1}\theta_{2}} & R_{\lambda_{2}\theta_{2}} & R_{\lambda_{3}\theta_{2}}\\ R_{\lambda_{1}\theta_{3}} & R_{\lambda_{2}\theta_{3}} & R_{\lambda_{3}\theta_{3}} \end{array}\right]\left[\begin{array}{c} P_{\lambda_{1}}\\ P_{\lambda_{2}}\\ P_{\lambda_{3}} \end{array}\right].$$

This relationship can be extended to a larger matrix. Let *λ_n_* be the wavelength range discretized into *n* components, *I_n_* be the current of the SPR peak value, *θ_n_* be the incident angle of the peak value, and the light intensity corresponding to each wavelength component *λ_n_* be *P_n_*. We obtain the following expression:

(4) $$\left[\begin{array}{c} I_{\theta_{1}}\\ \vdots \\ I_{\theta_{n}} \end{array}\right]=\left[\begin{array}{ccc} R_{\lambda_{1}\theta_{1}} & \cdots & R_{\lambda_{n}\theta_{1}}\\ \vdots & \ddots & \vdots \\ R_{\lambda_{1}\theta_{n}} & \cdots & R_{\lambda_{n}\theta_{n}} \end{array}\right]\left[\begin{array}{c} P_{\lambda_{1}}\\ \vdots \\ P_{\lambda_{n}} \end{array}\right].$$

In short, ***I*** = ***RP***, where ***P*** vector components indicate the intensity for each wavelength, and ***I*** vector components correspond to the measured photocurrents at each SPR angle of incidence. Since the diagonal components $R_{\lambda_{i}\theta_{i}}$ (1 ≤ *i* ≤ *n*) of the responsivity matrix ***R*** take a maximum among each column, the responsivity matrix ***R*** becomes regular with an inverse matrix. Because the ***P*** vector can be calculated by the equation ***P*** = ***R***^−^^1^***I***, the spectrum of the incident light can be derived.

When the operating wavelength range is narrow, the amplitude of the resonant angular position shift becomes narrow, and only a single SPR peak appears for each wavelength, as shown in a square of dotted lines in Figure 1c. If the wavelength range is expanded to cover the NIR wavelength range, the angular scanning range should also be expanded. Assuming that *λ*_3_ < *λ*_4_ < *λ*_5_, multiple SPR peaks corresponding to different diffraction orders *m* may appear in the scanning range, as shown in the solid line square in Figure 1c. For example, with respect to the response to *λ*_1_, not only *θ*_1_ but also *θ*_9_ peak appears. If we maintain a strategy to measure the photocurrents at SPR angles, the matrix takes the following form:

(5) $$\left[\begin{array}{c} I_{\theta_{1}}\\ \vdots \\ I_{\theta_{9}} \end{array}\right]=\left[\begin{array}{ccc} R_{\lambda_{1}\theta_{1}} & \cdots & R_{\lambda_{5}\theta_{1}}\\ \vdots & \ddots & \vdots \\ R_{\lambda_{1}\theta_{9}} & \cdots & R_{\lambda_{5}\theta_{9}} \end{array}\right]\left[\begin{array}{c} P_{\lambda_{1}}\\ \vdots \\ P_{\lambda_{5}} \end{array}\right],$$

so that the ***R*** matrix is not a square matrix. Therefore, it is impossible to perform a calculation using a simple inverse matrix. Therefore, as a simple extension, we adopted a method of constructing an ***R*** matrix using the measured current values at all SPR peak angles occurring in the angular scanning range and reconstructed the incident spectrum using the generalization inverse matrix. When a generalized inverse matrix is used, the spectral derivation can be applied for a wider range of wavelengths. In summarized form, the spectral derivation equations become:

(6) $$\left[\begin{array}{c} I_{\theta_{1}}\\ \vdots \\ I_{\theta_{k}} \end{array}\right]=\left[\begin{array}{ccc} R_{\lambda_{1}\theta_{1}} & \cdots & R_{\lambda_{n}\theta_{1}}\\ \vdots & \ddots & \vdots \\ R_{\lambda_{1}\theta_{k}} & \cdots & R_{\lambda_{n}\theta_{k}} \end{array}\right]\left[\begin{array}{c} P_{\lambda_{1}}\\ \vdots \\ P_{\lambda_{n}} \end{array}\right],$$

and

(7) $$P={\left(R^{T}R\right)}^{-1}R^{T}I,$$

where ***R***^T^ is a transpose of the ***R*** matrix. In the following, we experimentally constructed an ***R*** matrix and evaluated the applicability of the above method for NIR spectroscopy.

## 4. Materials and Methods

**Figure 2:**
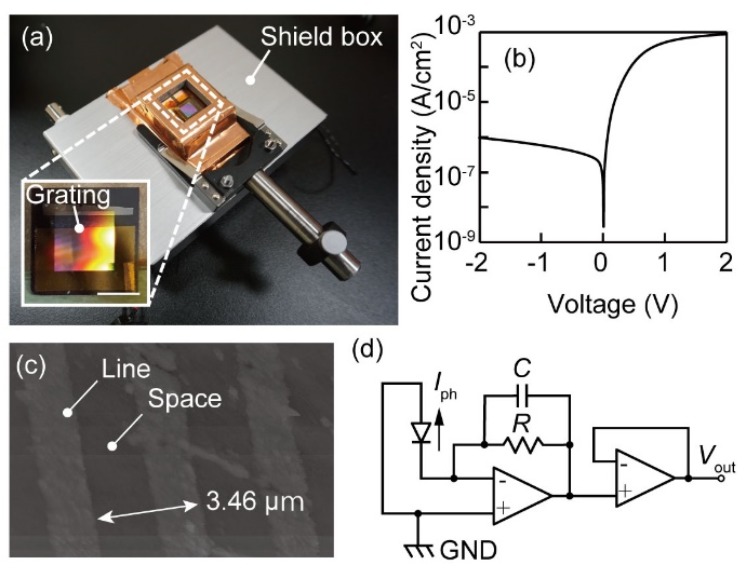
Photographs of the fabricated photodetector and the measurement circuit unit (bar = 5 mm). (**a**) The fabricated photodetector in a shield box. (**b**) A characteristic current-voltage curve of the photodetector. (**c**) An AFM image of the grating area. (**d**) An I-V conversion amplifier circuit.

The design of the photodetector device was slightly modified from that in the previous report [9]. The substrate of the device was an n-Si wafer whose resistivity was 10–20 Ω·cm. One-dimensional gratings with 3.46-μm-pitch (denoted as *a* in Figure 1a) and height *h* of 40 nm were formed on the front surface of the n-Si substrate with an area size of 12.7 mm × 12.7 mm by reactive ion etching. The surface had a 100 nm thick Au film caused by vacuum evaporation during rotation with an oblique angle such that the sidewalls of the grating were covered in gold. On the back side of the n-Si substrate, an Al film was also formed as a cathode electrode. A photograph of the device is shown in Figure 2a. To confirm the diode rectification and photodetection characteristics, the current-voltage curve was measured (Figure 2b). The curve presented a typical diode characteristic. The Schottky barrier height was calculated following a procedure described in Reference [16], and was 0.776 eV. Since the detection limit wavelength of the Schottky barrier height corresponds to 1.62 µm, near-infrared light detection was confirmed to be possible. In addition, a surface topographic image was taken using an AFM (JSPM-5000, JEOL, Tokyo, Japan). It was confirmed that a grating with a height amplitude of approximately 40 nm was fabricated. Since obtainable photocurrent signals for spectral measurements were on the order of nA to µA, the signal often suffered from noise. The current was therefore converted and amplified to a voltage in the immediate vicinity of the device using an operational amplifier to improve signal-to-noise ratio and signal resolution (Figure 2c). The first operational amplifier converted the output current *I*_ph_ to a voltage through a feedback resistor *R* = 1 MΩ according to an equation below:

(8) $$V_{o}=I_{\mathrm{ph}}\times R.$$

Then, the converted voltage *V*_o_ was passed to a voltage follower to provide the output voltage *V*_out_. In the following, the current values were calculated by dividing *V*_out_ by *R* = 1 MΩ. The conversion amplifier and the photodetector device were put into a shielding box, and an aperture was made in a box wall in front of the photodetector such that the light was incident on the photodetector, as shown in Figure 2a.

## 5. Experimental Results

**Figure 3:**
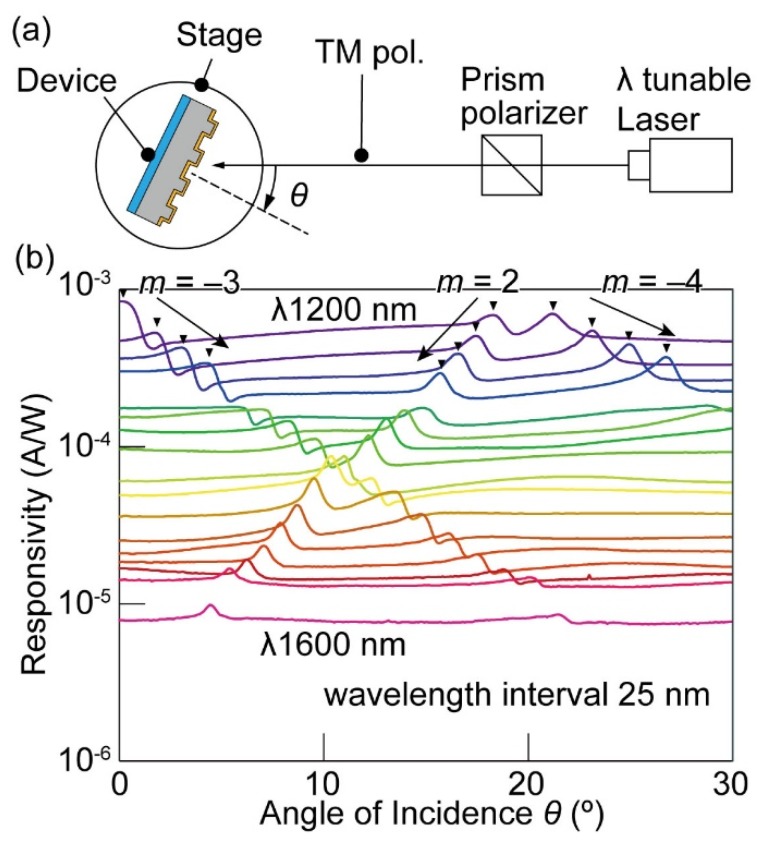
(**a**) An experimental setup. (**b**) Responsivity angular spectrum for near-infrared (NIR) monochromatic laser irradiation for wavelengths ranging from 1200 nm to 1600 nm.

A responsivity matrix ***R*** was experimentally constructed. The experimental setup is shown in Figure 3a. In the measurement, the device was fixed on a rotational stage, and infrared light from a wavelength tunable laser (SC-450, Fianium, Southampton, UK) was incident on the device. The wavelengths of the monochromatic infrared light were scanned from 1200 nm to 1600 nm with a 25-nm interval of wavelength. A linear polarizer was installed between the light source and the device such that the light became TM-polarized. The photocurrent was converted to a voltage signal by an I-V conversion amplifier and was measured using a source meter (6242, ADCMT, Tokyo, Japan). Using the rotating stage, the angle of incidence *θ* was rotated from 0° to 30° with a resolution of 0.1°. The light intensity at each wavelength was measured using a power meter (PM300, Thorlabs, Newton, NJ, USA). Experiments were conducted in a darkroom to prevent stray light. The obtained photocurrent angular spectra are shown in Figure 3b, where the SPR peaks are highlighted with arrows. The vertical axis is the logarithmic representation of the responsivity, and the horizontal axis is the angle of incidence. Since the laser spot area was smaller than the grating area, all of the incident energy was used to calculate the responsivity. Noise reduction due to the I-V amplifier provided clear photocurrent waveforms in a wide wavelength range. The SPR peak angular positions systematically shifted depending on the wavelength. Since the angular scanning range was wider than that in a previous report [9], multiple SPR peaks corresponding to different diffraction orders *m* were found in each curve. It was also found that a photocurrent was generated even when SPR did not occur. This baseline photocurrent can be attributed to excitation of electrons by the direct irradiation of the near-infrared light on the Au/n-Si Schottky interface through the Au film. This photocurrent presented a tendency to decrease with an increase in the wavelength because the photon energy of the light was inversely proportional to the wavelength. Elimination of this baseline photocurrent by optimization of device structures will be necessary to improve the spectrometer performance in the future.

**Figure 4:**
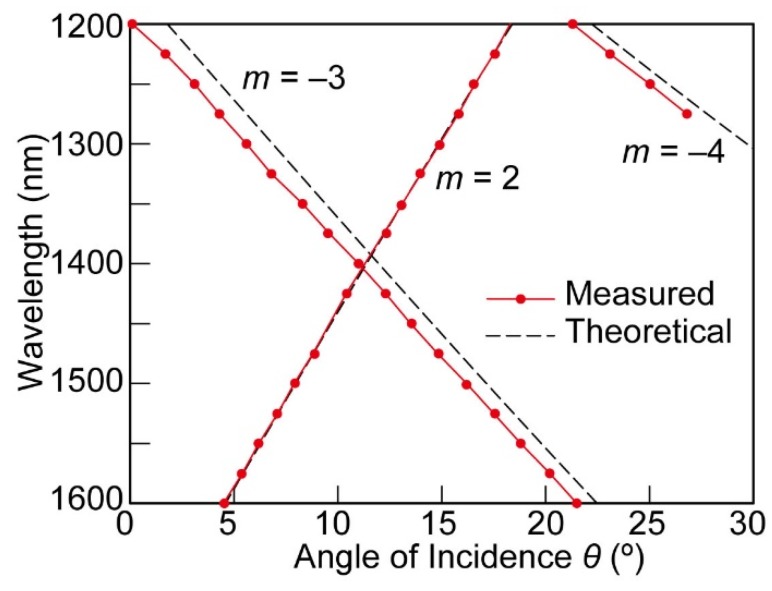
SPR angle positions with respect to the wavelength of the incident light. Measured and calculated theoretical angle positions belonging to three different diffraction orders.

To investigate the validity of the obtained angular spectrum, the SPR peak angular positions were compared with the calculated ones using Equation (1). The dispersive permittivity of the gold was taken from Reference [17] in the calculation. The calculated theoretical SPR angles were plotted with respect to the wavelength in addition to the measured angles in Figure 4. The calculated and experimental SPR angular positions showed high consistency. Although there was some error in the angle, particularly for *m* = −3, the amplitude was as small as 1°. It was therefore concluded that the obtained photocurrent peaks can be attributed to SPR. The responsivity matrix ***R*** was then constructed using responsivity values at SPR peak angular points belonging to all three diffraction orders.

**Figure 5:**
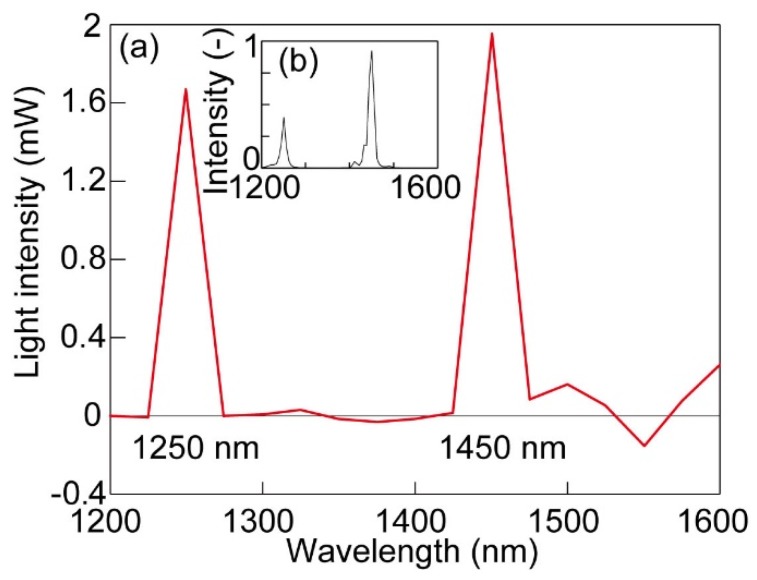
Spectrum of the light composed of two different wavelengths, 1250 nm and 1450 nm. (**a**) Calculated spectral data obtained with the fabricated device in this article, (**b**) spectrum of the light obtained with a commercial spectrometer.

To check the spectroscopy performance using an ***R*** matrix with a generalized inverse matrix method, light composed of two wavelengths, 1250 nm and 1450 nm, was irradiated at the same time, and the spectrum was calculated. The results are shown in Figure 5a.

The horizontal and vertical axes indicate the wavelength and intensity, respectively. Distinctive peaks were observed at *λ* = 1250 nm and 1450 nm. The intensity at these two peak wavelengths was consistent with the values measured with a power meter. The reference spectrum data in Figure 5b were measured with a commercially available NIR compact spectrometer (Sol. 2.2A, B&W Tek, Newark, DE, USA). Peak positions and spectrum shapes were consistent between the two. This spectral data consistency can be attributed to the fact that the inverse matrix method takes contributions from all diffraction orders into consideration. Therefore, NIR spectroscopy was performed using the responsivity data ranging from *λ* = 1200 nm to 1600 nm. There is, however, a difference on the power peak height between Figure 5a,b. It may be attributed to the laser intensity fluctuation because these two data were obtained at different times. Quantitative evaluation of the proposed sensor performance should further be performed in the future.

## 6. Conclusions

In this paper, we proposed NIR spectroscopy based on a Si photodetector equipped with a gold grating and extended the measurable NIR wavelength range by improving a spectrum derivation procedure from a previous study [9]. A responsivity matrix ***R*** was constructed, and a generalized inverse matrix of ***R*** was used to obtain the spectrum to fit a situation where multiple SPR peaks appeared among the scanning range. When light composed of two wavelengths, 1250 nm and 1450 nm, was irradiated at the same time, the two wavelengths were distinctively discriminated using the improved method. The reduction of the angular scanning resolution will provide a denser ***R*** matrix to improve the wavelength resolution. Moreover, since sharpening of an SPR peak curve shape can be realized by tuning the grating profile, the wavelength resolution can further be improved [18,19,20]. It is noted that the improvement of sensitivity should be pursued for the use with a normal white light source instead of a laser as a light source because the responsivity of the proposed device is still around 10–100 μA/W. Since the previous literature indicates that responsivity as large as several mA/W is possible with the similar plasmonic and Schottky approach [21], two-orders of improvement can be expected, which will provide us with spectrum data with practically high signal-to-noise ratio. The proposed spectrometer device is made of Si, so it is possible to integrate the angular scanning mechanism into the photodetector as a micro-electro-mechanical systems (MEMS) device [22,23]. The proposed spectroscopy method enables spectroscopy with only a thin-film plasmonic layer and an integrated photodetector located beneath the layer, and it is advantageous from the viewpoint of miniaturization. Advancement of the proposed method will provide a new microsized integrated spectrometer that will provide rich information about our environment.
